# Regulatory Evolution and Voltage-Gated Ion Channel Expression in Squid Axon: Selection-Mutation Balance and Fitness Cliffs

**DOI:** 10.1371/journal.pone.0120785

**Published:** 2015-04-13

**Authors:** Min Kim, Don McKinnon, Thomas MacCarthy, Barbara Rosati, David McKinnon

**Affiliations:** 1 Department of Neurobiology and Behavior, Stony Brook University, Stony Brook, New York, United States of America; 2 Institute of Molecular Cardiology, Stony Brook University, Stony Brook, New York, United States of America; 3 Department of Applied Mathematics and Statistics, Stony Brook University, Stony Brook, New York, United States of America; 4 Department of Physiology and Biophysics, Stony Brook University, Stony Brook, New York, United States of America; 5 The Department of Research, Veterans Affairs Medical Center, Northport, New York, United States of America; Dalhousie University, CANADA

## Abstract

It has been suggested that optimization of either axonal conduction velocity or the energy efficiency of action potential conduction predominates in the selection of voltage-gated sodium conductance levels in the squid axon. A population genetics model of channel gene regulatory function was used to examine the role of these and other evolutionary forces on the selection of both sodium and potassium channel expression levels. In this model, the accumulating effects of mutations result in degradation of gene regulatory function, causing channel gene expression to fall to near-zero in the absence of positive selection. In the presence of positive selection, channel expression levels fall to the lowest values consistent with the selection criteria, thereby establishing a selection-mutation balance. Within the parameter space of sodium and potassium conductance values, the physiological performance of the squid axon model showed marked discontinuities associated with conduction failure and excitability. These discontinuities in physiological function may produce fitness cliffs. A fitness cliff associated with conduction failure, combined with the effects of phenotypic noise, can account for the selection of sodium conductance levels, without considering either conduction velocity or metabolic cost. A fitness cliff associated with a transition in axonal excitability, combined with phenotypic noise, can explain the selection of potassium channel expression levels. The results suggest that voltage-gated ion channel expression will fall to low levels, consistent with key functional constraints, even in the absence of positive selection for energy efficiency. Channel expression levels and individual variation in channel expression within the population can be explained by regulatory evolution in combination with genetic variation in regulatory function and phenotypic noise, without resorting to more complex mechanisms, such as activity-dependent homeostasis. Only a relatively small region of the large, nominally isofunctional parameter space for channel expression will normally be occupied, because of the effects of mutation.

## Introduction

The voltage gated sodium and potassium channels expressed in the squid axon have characteristic average peak conductance values that have been established during the course of evolution. Two theories have been proposed to account for how the sodium conductance expression level may have been selected. It was initially suggested that the level of voltage-gated channel expression in the axon had been optimized in order to maximize action potential conduction velocity [1,2]. It was subsequently shown that sodium conductance values fall well below this optimum for conduction velocity and it was proposed that sodium channel expression levels had been optimized to minimize energy utilization during action potential generation [3,4]. Although it is conceivable that evolution has acted to perfectly optimize a single physiological property of the axon [5], it is likely that the effects of natural selection will be more complex [6,7].

Regulatory evolution refers to the evolution of protein expression levels, as opposed to structural evolution, which is the evolution of protein structure and function. For both theoretical [8,9] and practical reasons, a majority of experimental studies on regulatory evolution have focused on the evolution of cis-regulatory function [10,11]. Cis-regulatory evolution refers to the evolution of those regions of DNA within or in proximity to a given gene that determine mRNA expression levels from that particular gene. Cis-regulatory evolution has been established as a mechanism by which the expression levels of voltage-gated ion channels can change during the course of evolution [12,13]. In this respect, voltage-gated ion channels are similar to most major classes of proteins, whose expression levels appear to be primarily determined by the evolution of gene regulatory function [9,14]. Evolution of gene regulation is more flexible and consequently more common than evolution of protein structure, in part because it largely avoids the pleiotropic effects that can result from changes in the structure and function of proteins that can be expressed in many different cell types [8,9,15].

In this paper, a population genetics model [16] that incorporates a model of channel gene regulatory function was used to examine how ion channel expression levels in the squid axon could have been established and maintained during the course of evolution by selection-mutation balance. The primary focus is on understanding how purifying selection acts to stabilize expression levels at the observed values over successive generations and which physiological performance properties may be subject to natural selection.

## Methods

Two well established models of biological function, the Hodgkin and Huxley model of a propagating action potential in the squid axon [17] and the *K*-allele Wright Fisher model of population genetics [18], were combined in this analysis. The fitness and contribution to the next generation of individuals within the population model was determined by their physiological performance as described by the axon model.

### Axonal Conduction Model

The Hodgkin and Huxley model [17] of a propagating action potential in the squid axon was used to calculate the conduction velocity, the ability to conduct an action potential, ion fluxes per action potential and axonal firing properties over a broad range of peak potassium and sodium conductance values. Membrane potential was determined by the following system of equations,

$$\begin{array}{c} C_{m}\left(t\right)\frac{\partial V}{\partial t}=G_{a}\frac{\partial^{2}V}{\partial x^{2}}-G_{K}n^{4}\left(V-E_{K}\right)-G_{Na}m^{3}h\left(V-E_{Na}\right)-G_{L}\left(V-V_{L}\right)+I_{stim}/\left(2\pi r\right)\\ \frac{dn}{dt}=\alpha_{n}\left(V\right)\left(1-n\right)-\beta_{n}\left(V\right)n\\ \frac{dm}{dt}=\alpha_{m}\left(V\right)\left(1-m\right)-\beta_{m}\left(V\right)m\\ \frac{dh}{dt}=\alpha_{h}\left(V\right)\left(1-h\right)-\beta_{h}\left(V\right)h \end{array}$$

where *G*_*a*_ = *r* / (2*R*_*a*_). These equations were reformulated for a resting membrane potential of -65 mV and were solved numerically using the staggered backward Euler method of Hines [19], which is second order accurate in both time and space (*O*(Δ*t* ^2^, Δ*x* ^2^)). The simulated axon had length, *L =* 10 cm, axon radius, *r* = 238×10^−4^ cm, specific cytoplasmic resistivity, *R*_*a*_ = 0.0354 kΩcm, *G*_*L*_ = 0.3 mS / cm^2^, *E*_*Na*_ = 50mV, *E*_*K*_ = −77. *G*_*K*_, *G_Na_* and *V*_*L*_ were varied systematically in parameter sweeps (see below).

The model was implemented in Matlab (Mathworks), with Δ*t* = 1 μs and Δ*x* = 100 μm. Further increases in either the time or spatial resolution had no significant influence on the results. Simulations were performed assuming a temperature of 18.5°C, except where noted. A stimulus current of 20 μA for 0.1 ms was applied to the first compartment of the axon, except where noted. Once initiated, action potential conduction velocity was independent of the stimulus current.

The model incorporates a nonlinear membrane capacitance associated with the sodium channels (as described by Sangrey et al [3]). This was necessary to adequately model the dependence of conduction velocity on sodium conductance.

$$C_{m}\left(t\right)=C_{0}+C_{g}\left(t\right)$$

The total membrane capacitance *C*_*m*_(*t*) was given by the sum of a time invariant component, the intrinsic capacitance *C*_0_, and a time variable gating capacitance *C*_*g*_(*t*). The intrinsic capacitance *C*_0_ is a property of the lipid bilayer and had a value of 0.88 μF/cm^2^. The gating capacitance *C*_*g*_(*t*), is a property of the sodium channels and is linearly proportional to the number of closed sodium channels,

$$C_{g}\left(t\right)=0.13\frac{G_{Na}}{G_{Na,120}}\left(1-m\left(t\right)\right)\;\mu \text{F}/{\text{cm}}^{2}$$

where *G*_*Na*_ was the peak sodium conductance for a given simulation run and *G*_*Na*,120_ = 120 mS/cm^2^. The gating capacitance had a maximum value of 0.13 μF/cm^2^ for *G*_*Na*_ = 120 mS/cm^2^. For other sodium conductance values, the maximum gating capacitance was scaled relative to this value. Increasing the number of sodium channels increased the gating capacitance.

The rate equations were,

$$\begin{array}{c} \alpha_{n}=0.01\left(V+55\right)/\left(1-\text{exp}\left(-\left(V+55\right)/10\right)\right)\cdot Q_{10}\\ \beta_{n}=0.125\;\text{exp}\left(-\left(V+65\right)/19.7\right)\cdot Q_{10}\\ \alpha_{m}=0.1\left(V+40\right)/\left(1-\text{exp}\left(-\left(V+40\right)/10\right)\right)\cdot Q_{10}\\ \beta_{m}=4\;\text{exp}\left(-\left(V+65\right)/18\right)\cdot Q_{10}\\ \alpha_{h}=0.07\;\text{exp}\left(-\left(V+65\right)/20\right)\cdot Q_{10}\\ \beta_{h}=1.8/\left(1+\text{exp}\left(-\left(V+16\right)/10\right)\right)\cdot Q_{10}\\ Q_{10}=3^{\left(\left(T_{C}-6.3\right)/10\right)} \end{array}$$

An important variation from the original Hodgkin and Huxley model is the description of the potassium channel deactivation rate (*β*_*n*_), which was the same as that used by Clay et al [20]. The squid axon displays Type 3 excitability [20,21], meaning that it fires only once in response to a rapidly rising sustained depolarizing current stimulus, regardless of pulse duration [21,22,23,24]. This behavior is very different to the original Hodgkin and Huxley model, which has Type 2 excitability properties, meaning that it fires repetitively in response to a sustained depolarizing current with the firing rate remaining relatively independent of the stimulus strength [21,24]. The modified potassium channel kinetics results in greater activation of the potassium current in the subthreshold range [20], which is required to produce Type 3 excitability [23].

To determine the minimum sodium conductance required for action potential conduction in the model, some simulations were performed at 26°C. This is the maximum water temperature that *Loligo vulgaris* is likely to encounter during its life cycle [25]. Favored temperatures are lower, in the range 12.5–20°C [26]. The effect that varying potassium conductance has on excitability was determined at low temperature (6.3°C). Double firing of the axon in response to normal synaptic input is only observed at low temperatures, although this repetitive firing may reflect changes in the upstream motor pattern rather than an increase in axonal excitability [27]. When tested at other simulation temperatures the transition between the two firing behaviors proved to be relatively independent of temperature.

The specific leak conductance (*G*_*L*_ = 0.3 mS / cm^2^) was kept constant for all parameter sweeps of *G*_*K*_ and *G*_*Na*_. The leak conductance determines the ease with which the axon can be activated by synaptic inputs and the efficiency of the axon as an electrical cable. There is no reason to believe that the evolution of these properties would be directly linked to evolutionary mediated changes in voltage-gated channel expression. The leak potential was adjusted to give a stable resting membrane potential of -65 mV at the start of each simulation for any combination of sodium and potassium conductance values.

### Regulatory Evolution Model

The regulatory evolution models were adaptations of the *K*-allele Wright Fisher model [18]. This model assumes a fixed population of *N* diploid individuals where generations are non-overlapping and individuals are selected randomly without bias to mate. In the model every individual in the population was represented by two pairs of alleles, one pair determining sodium channel expression and one pair determining potassium channel expression, although for many simulations either the sodium or potassium channel alleles were fixed throughout the simulation.

#### K-allele model

The combined function of the cis-regulatory modules (CRMs) and promoter controlling expression of a given allele of either the voltage-gated sodium or potassium channel genes was summarized as a single value, representing the rate of mRNA expression from that allele. The range of possible values that a single allele, *a*, could take were the non-negative integers up to *K* – 1, the upper bound, so that *a* ∊ (0,1,2,3,…,*K* – 1). In the simulations without phenotypic noise, these values correspond directly to the peak conductance values that were used to simulate electrical activity using the model of axonal conductance described above. In the case of sodium channel expression, an allele strength of *n* corresponded to a peak sodium conductance of 5*n* mS/cm^2^ and in the case of potassium, 2*n* mS/cm^2^. The sodium channel conductances described by one allele were elements of (0,5,10,15,…,5(*K* – 1)) and the potassium channel conductances elements of (0,2,4,6,…,2(*K* – 1)). The two alleles for each conductance were summed to give the total sodium or potassium conductance. The upper bound, *K* – 1, was set sufficiently high that it was never reached during any simulation.

#### Extensions of the K-allele Wright-Fisher Model

The Wright-Fisher model was extended to describe the effect of mutation on gene regulatory function, the effect of selection based on physiological performance and the effect of phenotypic noise. Each of these extensions is described below.

#### Mutation Model

The effect of single mutations on regulatory function in the model was set to be small and constant since mutations with small, tissue-restricted effect are most likely to become fixed in a population [28]. A single mutation produced one step in either direction along the sequence of alleles. The effective mutation rate used in the simulations was *μ* = 1×10^−2^. Mutation effects were biased: 90% of mutations decreased promoter/CRM function whereas only 10% increased function (see below).

With this combination of mutation rate and effect size the simulations reached equilibrium within approximately 3000 generations (see Results). Significant evolution of the regulatory function of eukaryotes can occur within 300 generations [29] suggesting that the rate of regulatory evolution in the model is not unreasonably rapid. In prokaryotes, when all selective pressure for expression of a given enzyme is removed, expression levels fall to near zero levels within 400–500 generations [14]. The time course, measured in generations, for similar changes in channel expression in our model is approximately 7-fold slower (see Results), which is consistent with the greater complexity and more distributed nature of gene regulation in eukaryotes [30]. Different mutation rates or effect sizes changed the rate of approach towards equilibrium but had no significant effect on most results or the overall conclusions of this study. The mutation rate did affect the specific balance point in the mutation-selection equilibrium simulations, however, the interpretation of these results remained unchanged.

The directional bias of the effects of mutation in the model is based on experimental data. Structural constraints on the cis-regulatory modules and the promoters that control gene expression are generally less severe than they are for protein structure, which is one reason why these elements can evolve relatively easily. There is a bias in the effect of mutations, such that the majority of mutations decrease function, i.e. decrease transcription rates. High throughput saturation mutagenesis of specific regulatory elements gives an estimate of this bias [31,32,33]. The majority of single nucleotide mutations reduce the transcriptional activity of eukaryotic CRMs as expected, since random mutations will be more likely to eliminate or degrade transcription factor binding sites than to enhance binding or create new binding sites. For multiple different CRMs, 79% [31], 70% [32] or 81% [33] of single nucleotide mutations that produced a significant change in transcriptional activity were found to reduce enhancer activity, with the remainder producing an increase. Because mutations can decrease the binding affinity of both activating and repressive transcription factors in these experiments, the cumulative effect of mutations in reducing transcription factor binding and degrading regulatory function is likely to be larger than these values suggest. No consistent directional bias in the magnitude of the effect of mutation on transcriptional activity was observed, which is consistent with an effect on both activator and repressor binding. The effect of mutations on core promoters was even more strongly directional, primarily reducing transcriptional activity [34].

In the regulatory model, mutations were randomly assigned as decreasing or increasing collective promoter/CRM function. The probability that a mutation would reduce regulatory function was set at 0.9. The directional bias in the effect of mutations must be less than one or positive changes in regulatory function could not occur and is clearly significantly greater than 0.5, based on both the saturation mutation results and a commonsense understanding of promoter/CRM function. Within the range 0.7 to 0.95 the specific value selected had no significant effect on the results.

#### Selection Model

The selection model was based on physiological performance, as defined by the axon conduction model described above. Fitness functions, which are described in detail in the Results, reflected either qualitative or graded changes in physiological performance or a combination of both. Qualitative changes, such as the ability or inability to conduct an action potential were modeled as step changes in the fitness function thereby creating fitness cliffs. Graded changes in physiological performance, such as changes in conduction velocity, produced graded fitness functions. The fitness of an individual describes the probability of making a contribution to the next generation. If an individual does not pass the fitness test two new alleles are selected and the process repeats until *N* offspring survive in each cycle.

#### Noise Model

There are thought to be three main contributors to experimentally observed variations in protein expression between individuals within a population.

- Genetic variation in the gene’s cis-regulatory function (described above).
- Genetic variation in background genes. Background genes are those genes whose expression and function can act in *trans* to affect the expression of proteins from the gene in question.
- Phenotypic noise. This includes a variety of noise sources including biochemical, developmental and environmental noise [35,36], all of which can potentially affect the level of protein expression.

Individuals from a population with identical cis-regulatory genotypes for a given gene, will still display variation in protein expression levels from that gene due to the second and third noise sources. Because the squid is an outbred population that develops in a natural environment, the contribution of these other noise sources is likely to be significant. Background genetic variation and phenotypic noise were lumped together as a single noise source in the model, termed ‘phenotypic noise’ throughout. The magnitude of this phenotypic noise was constrained by experimental observation. Channel conductance values can vary up to four-fold in identified neurons from natural populations [37]. Phenotypic noise was modeled as a single binomial distribution centered on zero and was added to the phenotype predicted by a given genotype. The level of noise required to produce an adequate fit to the channel conductance levels observed in squid axon was determined by iteration. The spread of the 1 to 99 percentile of channel expression levels for a given channel type was constrained to be less than 4-fold during this process.

Simulations were run in both the presence and absence of phenotypic noise. In the absence of noise there was a linear mapping between genotype (transcriptional strength of a given allele) and phenotype (level of channel expression). In these cases, variation in channel expression was due solely to allelic variation in the cis-regulatory function of the voltage-gated sodium and potassium channel genes.

#### Simulation

The mutation and selection models described above can be incorporated into the standard K-allele Wright-Fisher model [18]. The population at generation *t* ∊ (0,1,2,3,…) is labeled *X*(*t*). Since we have *K* allele types it is convenient to write *X*(*t*) = (*X*_1_(*t*),*X*_2_(*t*),…,*X*_*K*_(*t*)) where *X*_*i*_(*t*) is the number of copies of allele type *i* in the population at time *t*. The sum of all alleles will be 2*N*, so that *X*_1_ + *X*_2_ + … + *X*_*K*_ = 2*N*. Fitness functions are labeled *f*, and they map the set of phenotypes (sums of allele strengths) to the interval [0, 1]. The model then determines a discrete-time Markov chain on *X*. The transition probabilities of this Markov chain depend on the particular model under consideration.

An expression for a model with one channel gene, incorporating mutation and selection but not phenotypic noise, is given. In this case, if the distribution of the population, *X*(*t*) = (*X*_1_(*t*),*X*_2_(*t*),…,*X*_*K*_(*t*)), at time *t* is (*m*_1_,*m*_2_,…,*m*_*K*_), then the probability that *X*(*t* + 1) is equal to (*n*_1_,*n*_2_,…,*n*_*K*_), is

$$\text{Prob}\left(X\left(t+1\right)=\left(n_{1},n_{2},\ldots ,n_{K}\right)|X\left(t\right)=\left(m_{1},m_{2},\ldots ,m_{K}\right)\right)=\frac{\left(2N\right)!}{n_{1}!n_{2}!\ldots n_{K}!}\overset{K}{\underset{i=1}{\prod }}{\left(\frac{a_{i}}{1-b}\right)}^{n_{i}}$$

where

$$\begin{array}{c} a_{i}=\frac{m_{i}}{2N}\left(1-\mu \right)E\left[f\left(i+R_{i}\right)\right]+0.9\frac{m_{i+1}}{2N}\mu E\left[f\left(i+R_{i+1}\right)\right]+0.1\frac{m_{i-1}}{2N}\mu E\left[f\left(i+R_{i-1}\right)\right]\\ b=\sum_{k=1}^{K}\frac{m_{k}}{2N}\left[\begin{array}{c} \left(1-\mu \right)\left(1-E\left[f\left(k+R_{k}\right)\right]\right)+0.9\mu \left(1-E\left[f\left(k-1+R_{k}\right)\right]\right)\\ +0.1\mu \left(1-E\left[f\left(k+1+R_{k}\right)\right]\right) \end{array}\right] \end{array}$$

*μ* is the mutation rate, *f* is the fitness function under consideration and *E*[*f*] is the expectation of *f*. *R*_*k*_ is a random variable representing the choice of the second allele in the genotype. It is distributed over the population at time *t* minus one copy of allele *k* after mutation. The constants 0.9 and 0.1 reflect the bias in the mutation direction. With the definition above, *a*_*i*_ is the probability that a copy of the *i*th allele is chosen for reproduction (either directly or by picking an allele that mutates into it) on a given iteration of the simulation and *b* is the probability that some allele is picked but does not pass the fitness test.

Although transition probabilities for the Markov chain can be described for most of the models used in this paper these probabilities cannot be used to efficiently compute successive generations due to their complexity. As a consequence, it was necessary to use a brute-force iterative approach for the simulations.

**Fig 1:**
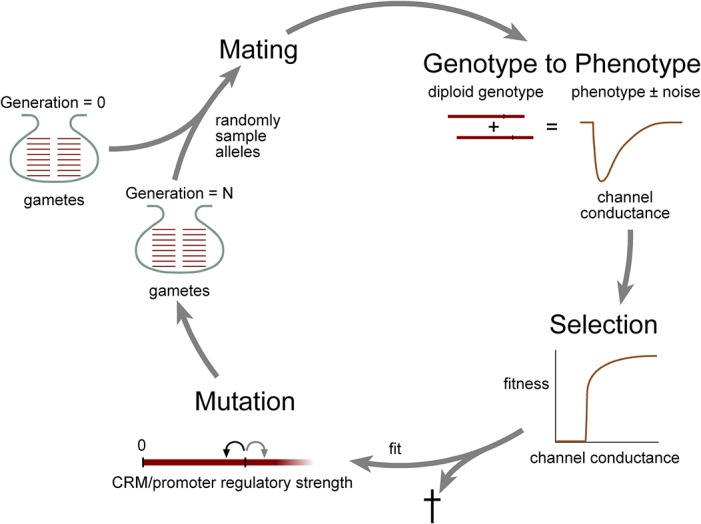
Schematic representation of the regulatory evolution model. The phenotype (channel peak conductance) of each individual was determined by random selection of two alleles from a pool of alleles (gametes). Channel conductance was directly proportional to the sum of the strength of the two alleles (see Methods), with or without a component of phenotypic noise. Fitness functions based on axonal electrophysiological performance were used to determine which individuals contributed genetic material to the next generation. This cycle repeats indefinitely. Random mutation of gamete DNA (channel gene promoter/CRM function) was biased towards a reduction in regulatory strength (reduced rate of mRNA transcription). In the simulations, mutation was performed immediately before selection in order to minimize computational costs but the results are equivalent to the natural order, where mutations would occur during gamete formation, as shown in the figure.

In the model each generation completes the cycle shown in Fig 1: individuals are selected to mate, offspring genotypes are converted to phenotypes (either directly or with noise), offspring are subjected to a survival test determined by their fitness, offspring alleles are mutated with mutation rate, *μ*, and the resulting genes are propagated to the next generation.

At the beginning of the simulation, an initial population of size *N* was generated. The allele distribution in the initial population had minimal effect on the final results. Typically, the alleles in the starting population were varied around an average value to model preexisting genetic variation. This variation was modeled as a binomial distribution (*p* = 0.5,*N* = 6) i.e. $\text{P}\left(a\right)=\left(\begin{array}{c} N\\ a \end{array}\right)p^{a}{\left(1-p\right)}^{N-a}$, which was centered on the starting average allele value. To build each new generation of *N* individuals allele pairs, *i* and *j*, were randomly sampled without replacement (i.e. *i* ≠ *j*) from the previous generation to form (*i*,*j*), the diploid zygote genotypes of the next generation.

The population size, *N*, in most simulations was 5000. Little is known about the effective breeding population size of squid but this parameter did not have a critical effect on the results. There was a small (approximately 2.7 mS/cm^2^) increase in final average sodium conductance with each 10-fold increase in population size (see Results). Data from the simulations are presented as means and standard deviation (S.D.) where appropriate.

## Results

### Model of the Evolution of Channel Gene Regulatory Function

An outline of the model of sodium and potassium channel gene regulatory evolution is shown in Fig 1 (see Methods for details). In the model, each individual has two alleles for each channel, which are inherited independently (only one set of alleles for one channel are shown in the figure). These alleles vary only in their regulatory function (the level of channel expression), channel biophysical function was assumed to be invariant over the time course of the simulations. The level of channel expression in the axon was a linear sum of the values of these two alleles with or without a contribution of phenotypic noise. Each individual was subject to a test of fitness, which was defined probabilistically by fitness functions based on the physiological performance of their axon. Individuals favored by selection contributed their genetic material to the next breeding cycle. This generational cycle could repeat indefinitely.

### Physiological Performance Properties of the Squid Axon that May Contribute to Fitness

**Fig 2:**
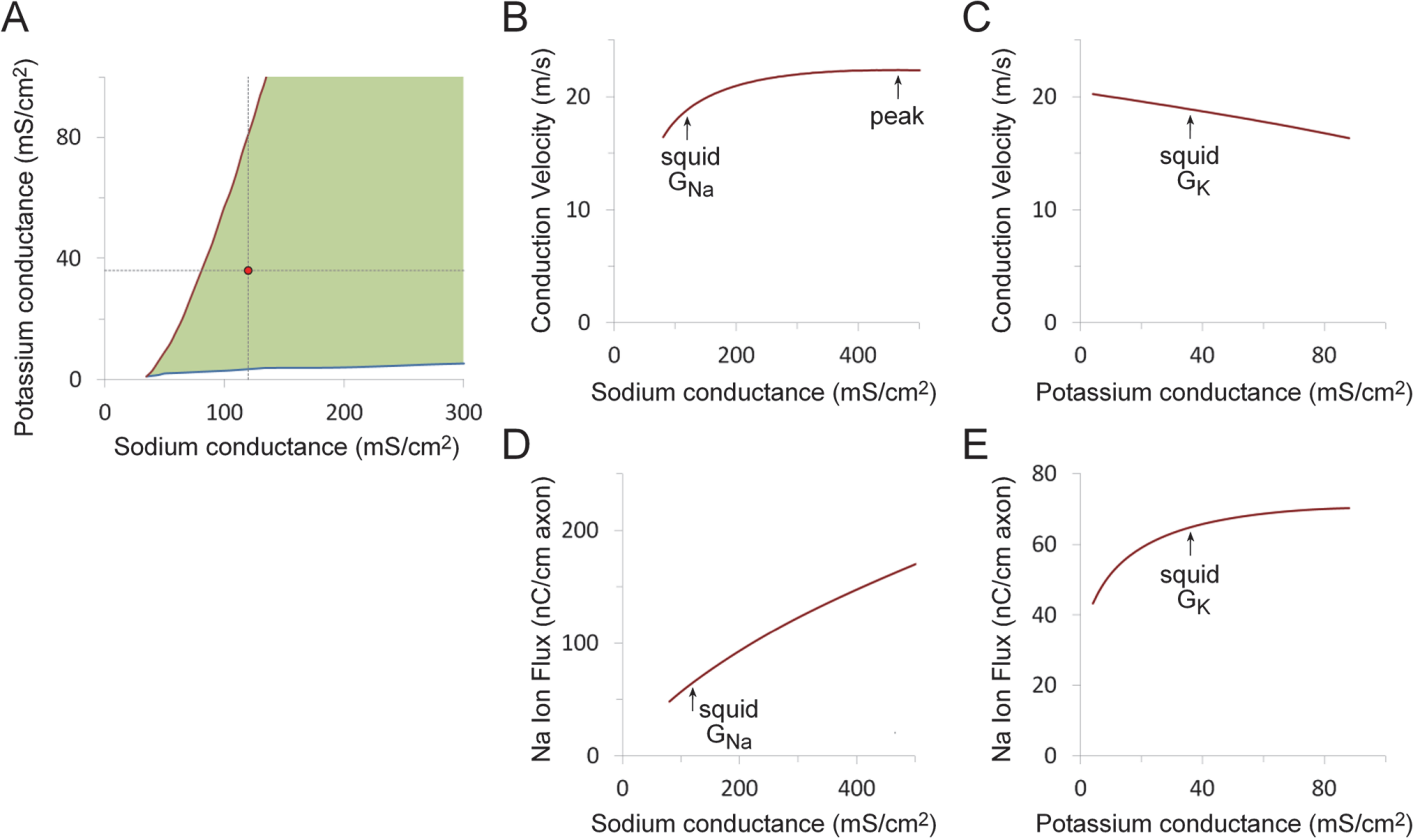
Effect of variations in sodium and potassium channel expression on physiological function. A. Region of sodium and potassium conductance parameter space that supports action potential conduction up to 26°C (filled green area). Upper (red) line corresponds to boundary above which action potential conduction fails to propagate. Lower (blue) line corresponds to boundary below which the action potential fails to repolarize. The experimentally observed combination of conductance values is marked with a red dot. B. Dependence of conduction velocity on peak sodium conductance. The sodium conductance at which conduction velocity peaks (465 mS/cm^2^) is marked with an arrow (labeled ‘peak’), as is the experimentally observed value (120 mS/cm^2^) (labeled ‘squid G_Na_’). C. Dependence of conduction velocity on peak potassium conductance. The experimentally observed value (36 mS/cm^2^) is marked (labeled ‘squid G_K_’). D. Dependence of sodium ion flux during the action potential on sodium channel conductance. E. Dependence of sodium ion flux during the action potential on potassium channel conductance. For the simulations shown in panels B-E, one voltage-gated conductance was kept constant (either potassium conductance = 36 mS/cm^2^ or sodium conductance = 120 mS/cm^2^), while the other conductance was swept over the range of conductance values for which action potential conduction did not fail at 26°C. The leak conductance was kept constant (0.3 mS/cm^2^) for all simulations. The simulation temperature was 18.5°C.

There are several physiological properties of the squid axon that can potentially contribute to fitness. The most fundamental of these is the ability to reliably transmit an action potential along the length of the axon. In squid, action potential propagation fails as the temperature increases [38]. The maximum temperature *Loligo vulgaris* is likely to encounter in vivo is 26°C [25,26] and tests of conduction failure were performed at this temperature. A broad range of sodium and potassium conductance combinations are compatible with action potential conduction (Fig 2A).

At the experimentally observed potassium conductance of 36 mS/cm^2^, conduction failure occurs when the sodium conductance falls below 81 mS/cm^2^ (Fig 2A). This provides a reasonable safety margin since the experimentally observed conductance value is 120 mS/cm^2^. At most temperatures the squid might encounter, the safety margin would be significantly larger because conduction becomes more robust as the temperature falls. There is no upper limit for sodium channel expression with respect to action potential generation because action potential conduction is reliable for all higher sodium conductance values. At 26°C and a sodium conductance of 120 mS/cm^2^, conduction fails at very low potassium conductances (below 3 mS/cm^2^), where the axon fails to repolarize, and at relatively high potassium conductances (above 88 mS/cm^2^), where it cannot maintain a self-sustaining action potential (Fig 2A).

A failure to generate a propagating action potential in the giant axon is likely to produce a dramatic reduction in fitness due to significant impairment of both predator escape and prey-capture behaviors [39,40]. It is conceivable that a squid with an unlucky combination of alleles that does not support action potential conduction could adjust channel expression homeostatically in order to maintain conduction. Notably, however, there is no evidence for homeostatic regulation of these channels in the squid axon. Seasonal changes in channel conductances, which could adapt axonal physiology to changes in environmental temperature, might confer some survival advantage for this species, yet they do not occur [41]. As a consequence, conduction failure due to deleterious combinations of allelic channel regulatory function will almost certainly be addressed at the population level by reduced survival, as occurs for other genetic disorders affecting channel expression [42].

The large diameter of the giant axon suggests that this anatomical trait is subject to strong selection for increased conduction velocity and selection for increased conduction velocity might also influence axonal physiology. As has been described previously [1,2,3], conduction velocity first increases with increasing sodium conductance, plateaus, and then begins to decline at higher sodium conductance values. Only the range of conductance values over which conduction velocity increases are shown (Fig 2B), since it would be unlikely for natural selection to select sodium channel expression values that both reduced conduction velocity and increased metabolic cost. Increasing sodium conductance levels, in addition to increasing the depolarizing sodium current, which increases conduction velocity, also increases the sodium channel gating charge, which acts as a nonlinear capacitance that slows conduction velocity at higher conductance levels [1,2]. As originally noted by Levy and associates [3], the sodium conductance at the peak conductance velocity value is much higher than the experimentally observed value (Fig 2B). It is notable, however, that the relationship between sodium conductance and conduction velocity in this region has a broad plateau. Although there is a large difference (74%) between the observed sodium conductance and the conductance at the peak conduction velocity, there is only a modest difference (16%) in the maximum conduction velocities at these two values. Conduction velocity declines steadily with increasing potassium conductance (Fig 2C).

The metabolic cost of action potential generation could potentially contribute to fitness [4,43,44]. Metabolic cost is directly proportional to the magnitude of the sodium ion flux across the cell membrane per action potential firing. This flux increases roughly linearly with increasing sodium conductance (Fig 2D). Ion flux per action potential firing also increases as the potassium conductance increases (Fig 2E).

As discussed below, these physiological properties of the action potential cannot adequately explain how the observed potassium conductance might be selected. The potassium channel also controls the subthreshold behavior of the axon [20,21,23,24]. The mantle of the squid produces an all-or-none contraction in response to a single firing of the giant axon [39,45] and only a single firing occurs during short latency escape responses [46]. Given the dramatic behavioral consequences of axon firing it is not surprising that this has evolved to be a relatively inexcitable, low noise system. One index of the low excitability of the giant axon is the observation that the axon fires only a single action potential in response to a sustained depolarizing current before becoming silent [20,21]. This property is known as Type 3 excitability [21,22,23,24] and is conserved in other squid species [21].

**Fig 3:**
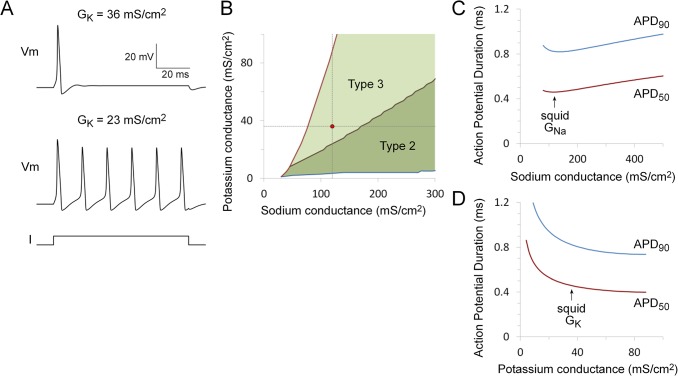
Effect of variations in sodium and potassium channel expression on firing patterns and action potential duration. A. Effect of reducing potassium conductance on excitability. At the normal potassium conductance of 36 mS/cm^2^, the axon model displays Type 3 excitability properties and is refractive after firing a single action potential in response to a sustained depolarizing current step. The axon is converted to Type 2 excitability and begins to fire repetitively following a reduction in potassium conductance (23 mS/cm^2^). The stimulus current was twice the size of the just-threshold current required to trigger an action potential and the sodium conductance was held constant at 120 mS/cm^2^. B. Region of sodium and potassium conductance parameter space that has Type 3 (light green area) or Type 2 (dark green area) firing properties. C. Dependence of action potential duration on peak sodium conductance. Action potential duration at 50% (red) and 90% (blue) of peak height are shown (APD50 and APD90, respectively). D. Dependence of action potential duration on peak potassium conductance. For the simulations shown in panels C and D, one voltage-gated conductance was kept constant (either potassium conductance = 36 mS/cm^2^ or sodium conductance = 120 mS/cm^2^), while the other conductance was swept over the range of conductance values for which action potential conduction did not fail at 26°C (Fig 2A). The leak conductance was kept constant (0.3 mS/cm^2^) for all simulations. The simulation temperature was 18.5°C for panels C and D and 6.3°C for panel A (see Methods).

Axonal excitability is strongly dependent on the magnitude of the potassium conductance. At the normal potassium conductance the axon is strongly refractory following the initial action potential (Fig 3A). This changes as the potassium conductance falls. For a fixed sodium conductance of 120 mS/cm^2^ there is a transition to Type 2 excitability with repetitive firing when the potassium conductance falls below 24 mS/cm^2^ (Fig 3A). The sodium and potassium conductance parameter space has a region with Type 3 excitability corresponding to relatively high potassium conductance and a region with Type 2 excitability properties corresponding to relatively low potassium conductances (Fig 3B).

Action potential duration is another property that can be constrained during the course of evolution [12]. Action potential duration is dependent on both sodium and potassium conductances (Fig 3C and 3D).

### Purifying Selection and the Establishment of Average Sodium Conductance Values

A goal of these simulations was to understand which physiological properties of the axon might be subject to purifying selection. Purifying selection (also known as negative selection) is the process by which deleterious alleles for a given gene function are eliminated from the population due to the negative effect of these alleles on individual fitness. In this example, purifying selection would act to maintain channel expression at stable levels over successive generations.

**Fig 4:**
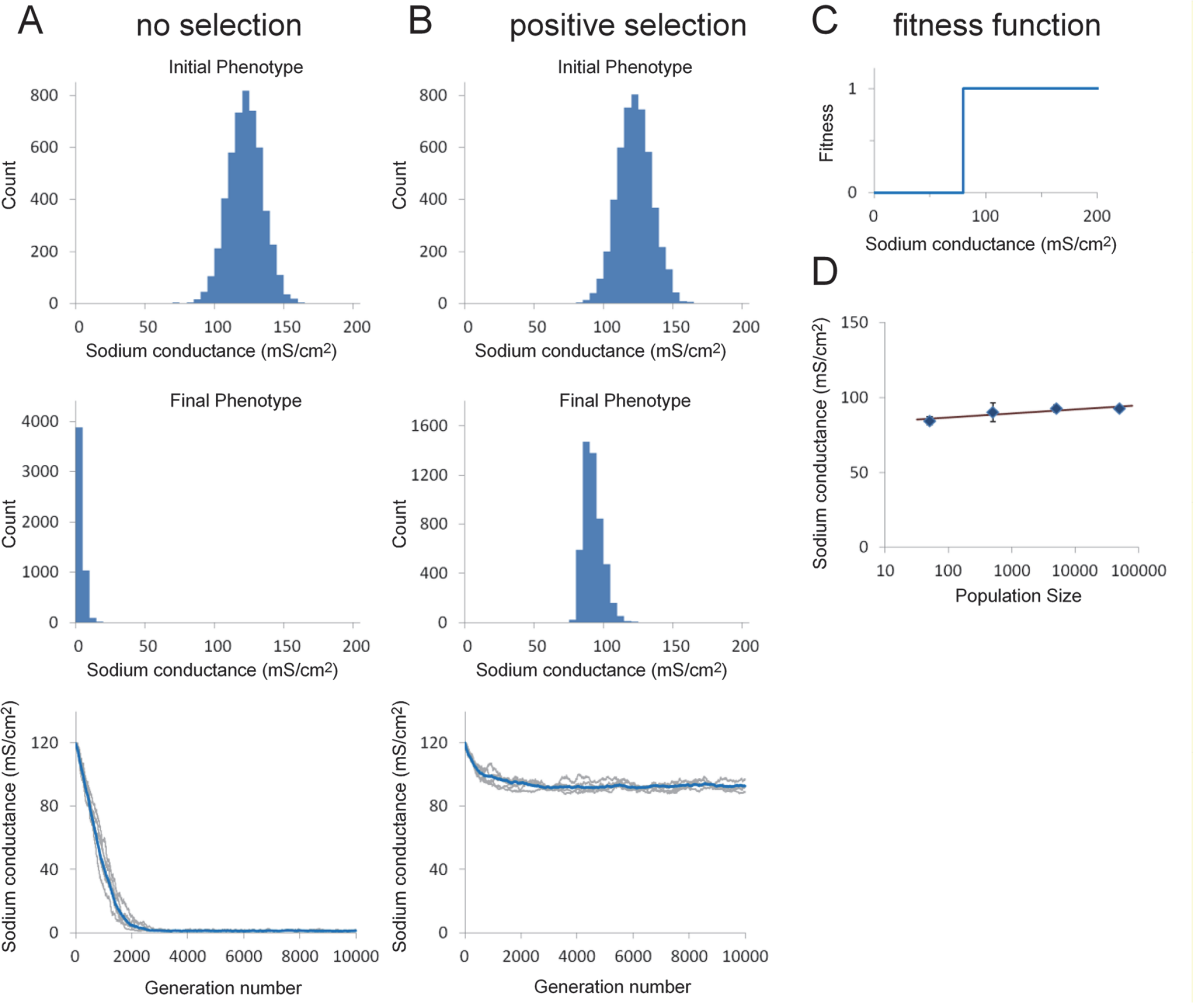
Effect of selection for action potential conduction on sodium channel expression over successive generations. A. Evolution of sodium conductance phenotype in the absence of any selection over 10000 generations (population size = 5000). Histograms show starting (top) and final (middle) phenotype distribution. The bottom panel shows how the average sodium conductance within the population evolves over time (10000 generations). The blue line is the average value of ten simulation runs and five independent runs are shown in light grey on the same graph. The average final conductance value was 1.2 ± 0.1 mS/cm^2^ (mean ± S.D., n = 10). It takes approximately 3000 generations to reach steady-state. B. Evolution of sodium conductance phenotype in the presence of positive selection, based on the fitness function shown in (C). Other parameters are the same as in (A). Average final conductance value was 92.7 ± 2.21 mS/cm^2^. C. The fitness function used in (B). This was based on the minimum sodium conductance required to maintain conduction along the axon at a temperature of 26°C. The plateau value of the step fitness functions has no significant effect on the results and a value of 1 was chosen for computational efficiency. D. Dependence of mean conductance on population size for the model shown in (B). Each ten-fold increase in population produced a 2.7 mS/cm^2^ increase in average conductance. Data points are means of ten simulation runs and error bars are S.D.

The starting genotype distribution was chosen so that the average sodium conductance phenotype of the starting population was centered on a conductance of 120 mS/cm^2^ (Fig 4A). The combined strength of each of the two alleles controlling sodium channel expression determined the phenotype for each individual within the population. There was random variation in allele strength within this initial population, which is reflected in variation in the initial channel expression levels (Fig 4A, top panel).

In the absence of any selection, the sodium conductance steadily falls to negligible levels over successive generations (Fig 4A, middle and bottom panels). The accumulating effect of mutations produces a steady decrease in the strength of the promoter and cis-regulatory modules (CRM) controlling sodium channel expression because deleterious mutations (mutations that reduce promoter/CRM function) are not removed from the population by purifying selection.

The simplest potential positive selection criterion for the sodium channel requires that the axon can support a propagating action potential at all likely environmental temperatures (Fig 2A). A fitness function based on this criterion is a step function with a value of zero for phenotypes with a sodium conductance below a minimum required for action potential propagation (Fig 4C). Using this criterion, the sodium conductance phenotype of the population piles up against this fitness cliff (Fig 4B, middle panel). The average conductance value under these conditions was 92.7 ± 2.21 mS/cm^2^ (mean ± S.D., n = 10), which is significantly lower than the experimentally determined value of 120 mS/cm^2^ [17].

Since this simple model could not account for the experimentally observed sodium conductance value, it was necessary to consider more complex models. There are three additional mechanisms, each of which when added to this basic model can act to stabilize the average conductance value at the initial value of 120 mS/cm^2^ over multiple generations.

The first mechanism incorporates the effect of the various sources of biological noise that will act to increase the variation in channel protein expression levels in individuals with equivalent channel regulatory genotypes (see Methods). The squid is a natural outbred population and there will be considerable genetic variation at all gene loci within the population. This background genetic variation will alter the efficiency with which the specific sodium and potassium channel cis-regulatory genotypes are translated into channel protein expression levels in an essentially random fashion so that individuals with identical channel regulatory genotypes will demonstrate variation in channel expression levels. In addition, there are multiple sources of noise that will cause variation in the channel protein expression phenotype even for individuals that have identical genomes. These noise sources include developmental noise, stochastic variation in the expression of mRNA and protein molecules between identical cells, variation of mRNA and protein expression within the same cell over time and the effects of environmental noise [35,36]. These different noise sources were treated collectively as single source, referred to as phenotypic noise throughout, that was introduced at the point of translation of each individuals genotype into the channel expression phenotype (Fig 1).

**Fig 5:**
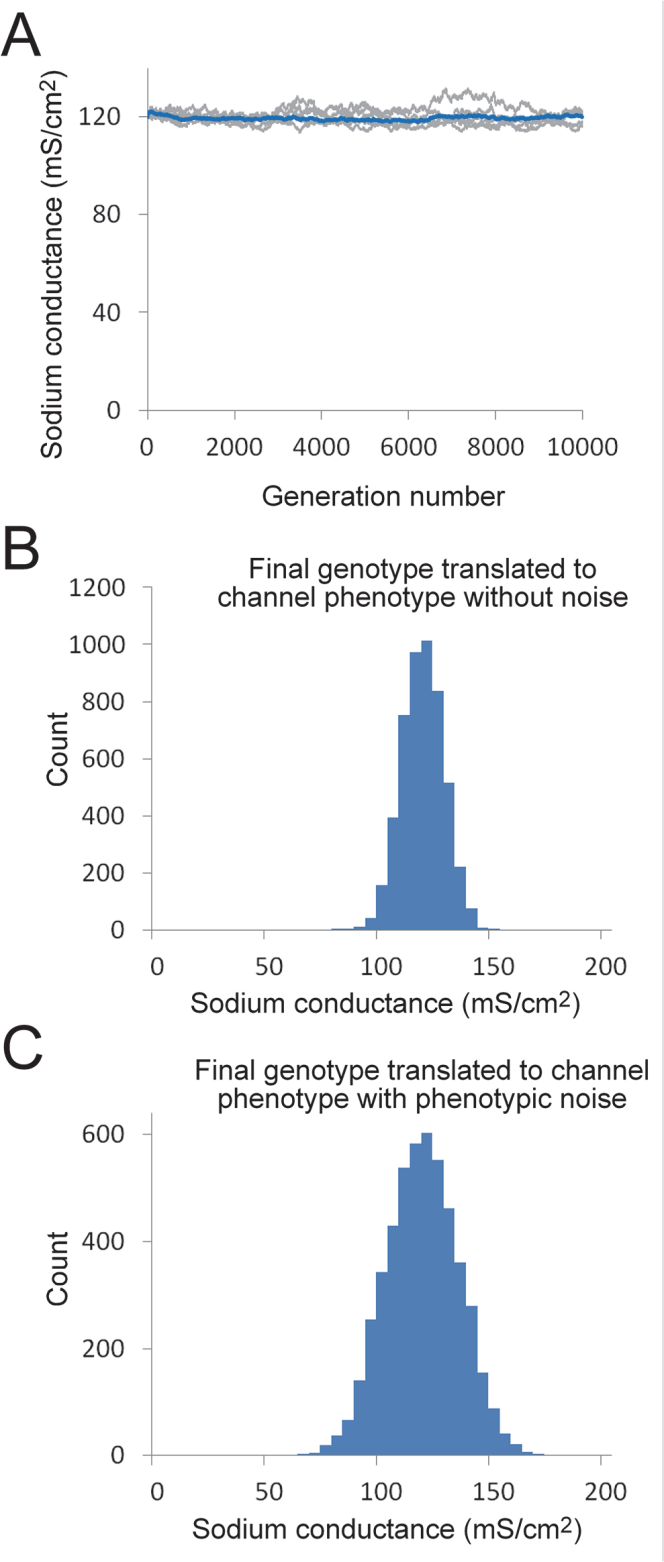
Effect of selection for action potential conduction on sodium channel expression over successive generations in the presence of phenotypic noise. A. Evolution of sodium conductance phenotype with the fitness function shown in Fig 4C in combination with phenotypic noise. The average sodium conductance within the population remains stable over time (10000 generations, population size = 5000). The blue line is the average value of ten simulation runs and five independent runs are shown in light grey on the same graph. The average final conductance value was 120.4 ± 4.4 mS/cm^2^ (mean ± S.D., n = 10). B. Histogram showing representative final phenotype distribution translated directly from the final genotype without the addition of phenotypic noise, in order to show the variation in the underlying sodium channel cis-regulatory genotype. C. Histogram showing representative final phenotype distribution, including the effect of phenotypic noise. This is the distribution of sodium channel protein expression values that was subject to selection. Phenotypic noise was modeled as a binomial distribution (N = 28) with an expected standard deviation of 13.2 mS/cm^2^. The N value for the noise distribution was fitted by iteration of the simulations in order to stably maintain the average conductance value close to the starting value. Note that the distributions in (B) and (C) come from the same simulation run.

The combination of a fitness cliff and phenotypic noise can maintain a stable sodium conductance indefinitely (Fig 5A), producing a final average sodium conductance of 120.4 ± 4.4 mS/cm^2^ (mean ± S.D., n = 10) after 10000 generations. The spread in the conductance values in the final population (Fig 5C) is due to two sources of variation, variation in the sodium channel cis-regulatory genotype (shown in Fig 5B) plus variation due to the effect of phenotypic noise. The range of conductance values in the final population is comparable to the two to four-fold ranges of channel conductance values that have been observed in identified neurons from natural populations [37], where both genotype variation and other noise sources would also be expected to contribute to variation in channel protein expression. Repeated iteration of the simulations was required in order to fit the appropriate noise distribution that could maintain a particular stable conductance value. Lower phenotypic noise levels produced lower average final conductance values and higher noise levels resulted in higher conductance values.

**Fig 6:**
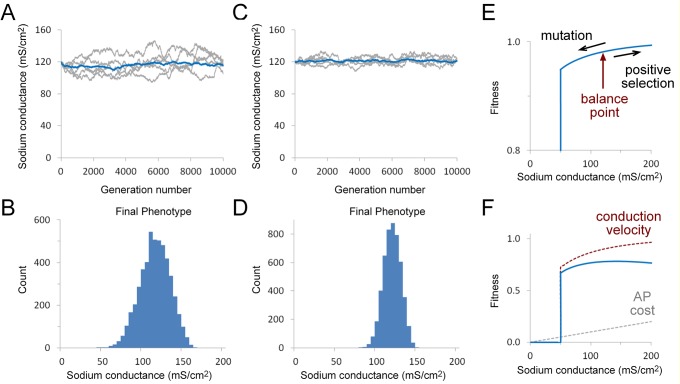
Effect of different selection criteria on sodium channel expression over successive generations. A. Evolution of average sodium conductance phenotype under selection for conduction velocity over time (10000 generations, population = 5000). The blue line is the average value of ten simulation runs and five independent runs are shown in light grey on the same graph. The average final conductance value was 116 ± 9 mS/cm^2^ (mean ± S.D., n = 10). B. Histogram of representative final phenotype distribution for a single simulation run. C. Evolution of average sodium conductance phenotype under selection for both conduction velocity and sodium conductance cost over time (10000 generations, population = 5000). The blue line is the average value of ten simulation runs and five independent runs are shown in light grey on the same graph. The average final conductance value was 120 ± 3.5 mS/cm^2^ (mean ± S.D., n = 10). D. Histograms of representative phenotype distribution for a single simulation run. E. Fitness function used in (A) and (B). Conduction as a function of sodium conductance (shown in Fig 2A) was scaled and combined with the fitness cliff shown in Fig 4C. Note the magnified scale. F. Fitness function (blue line) used in (C) and (D) was created by subtracting a cost of action potential generation (grey dotted line) from the conduction velocity (red dotted line).

The other two mechanisms that will allow purifying selection to maintain a stable sodium conductance phenotype require the incorporation of positive selection for conduction velocity into the fitness function. The first of these approaches assumes that increasing sodium conductance is cost-free so that the fitness function will reflect a scaled version of the conductance-velocity curve (Fig 6E). In the second approach, positive selection for conduction velocity is combined with a cost for increasing sodium conductance (Fig 6F).

Incorporating a contribution of conduction velocity together with a fitness cliff due to conduction failure can successfully maintain the average sodium conductance close to the initial phenotype (Fig 6A). This is an example of the classic mutation-selection balance that is believed to underlie the establishment of a broad range of phenotypic properties [16]. A balance is established between positive selection for increasing conduction velocity and the negative effect of mutation (Fig 6E). Because neither positive selection nor mutation have very strong effects on genotype under these particular conditions (note the magnified scale in Fig 6E), the effect of random drift becomes quite prominent so that the population mean shows considerable variation over time (Fig 6A) and the distribution of the final regulatory genotype/phenotype is relatively broad (Fig 6B). A steeper fitness curve, implying stronger selection for conduction velocity, results in a higher average conductance value as the conductance average is drawn closer to the conductance that produces peak conduction velocity (data not shown).

A fitness function incorporating an energy cost for action potential generation is shown in Fig 6F. The fitness cost for the ion fluxes was approximated by assuming that the energetic cost increases linearly with sodium channel conductance, which is an approximation of the results from the axon model (Fig 2D). In this case, increasing conduction velocity (red line) is now balanced by increasing cost (grey line) so that there is a distinct peak in the fitness function (blue line). This form of the fitness function can also maintain the average conductance values close to the starting phenotype over multiple generations (Fig 6C). Because the slopes of the fitness curve are now steeper, the effect of random drift is diminished and both the time courses of independent trials and final phenotypes have a tighter distribution (Fig 6C and 6D). In this case, the balance between the two competing selection constraints is the primary determinant of the final phenotype, although mutational effects will still cause the average conductance to fall below the peak of the fitness function.

### Purifying Selection and the Establishment of Average Potassium Conductance Values

The approach for understanding how purifying selection may maintain stable potassium conductance values was similar to that used for the sodium conductance. As with the sodium channel, evolution in the absence of any positive selection criteria results in almost complete elimination of potassium channel expression (G_K_ = 2.2 ± 0.8 mS/cm^2^ after 10000 generations). A fitness cliff based solely on the ability of the axon to reliably support action potential conduction up to 26°C (potassium conductance ≥ 3 mS/cm^2^) also results in selection of a very low average conductance value (G_K_ = 9.2 ± 0.8 mS/cm^2^ after 10000 generations) and is clearly inadequate to explain the observed conductance of 36 mS/cm^2^. Selection for increased conduction velocity or reduced metabolic cost will only act to reduce the potassium conductance (Fig 2C and 2E), suggesting that neither of these properties can make a significant contribution to the fitness function for the potassium conductance.

**Fig 7:**
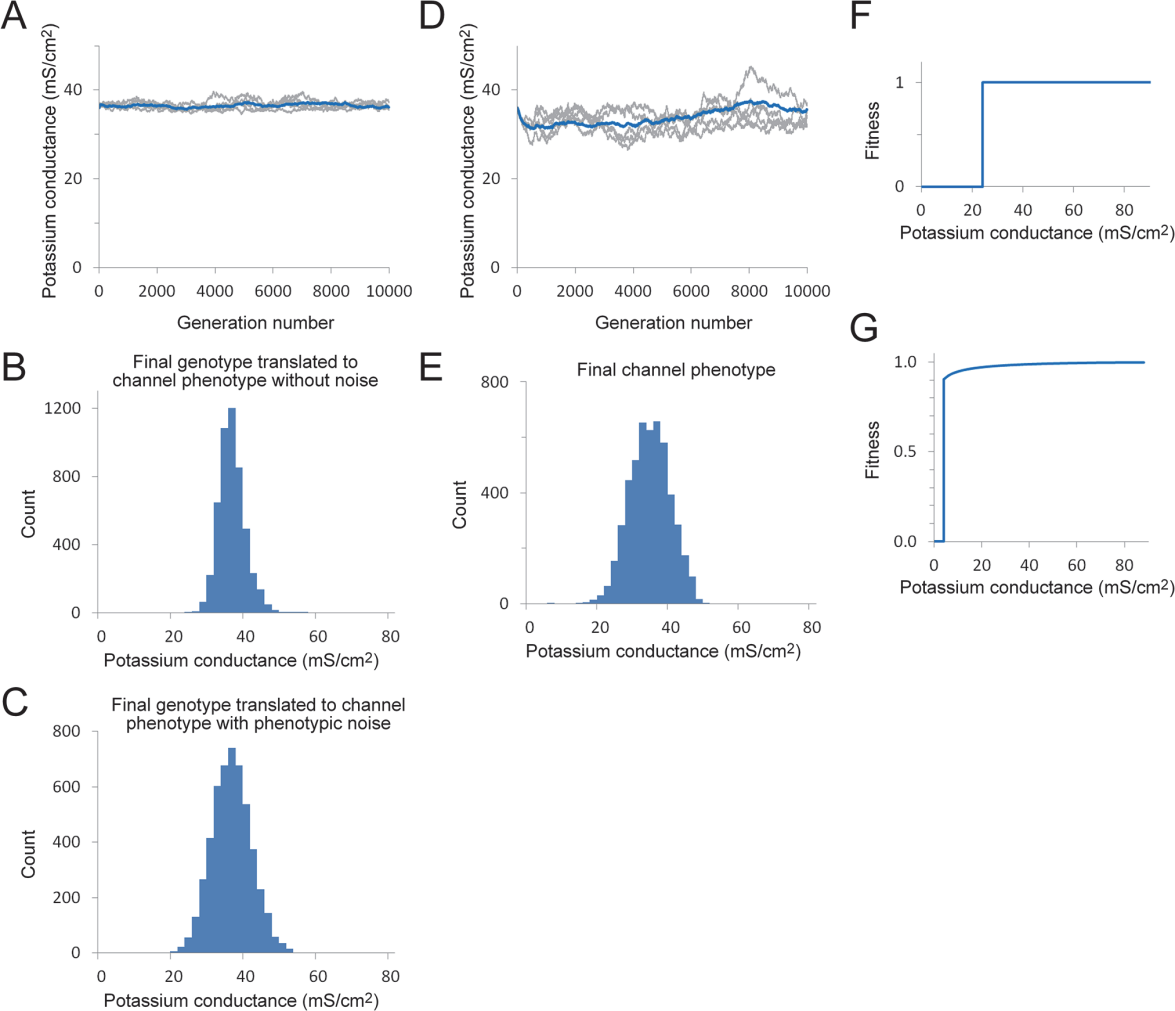
Effect of different selection criteria on potassium channel expression over successive generations. A. Evolution of potassium conductance phenotype with the fitness function shown in Panel F in combination with phenotypic noise. The average potassium conductance within the population remains stable over time (10000 generations, population = 5000). The blue line is the average value of ten simulations and five independent runs are shown in light grey on the same graph. The average final conductance value was 36.2 ± 0.5 mS/cm^2^ (mean ± S.D., n = 10). B. Histogram showing representative final phenotype distribution translated directly from the final genotype without phenotypic noise to show the variation in the underlying potassium channel genotype. C. Histogram showing representative final phenotype distribution, including the effect of phenotypic noise. It is this distribution of potassium channel protein expression values, which varies slightly with each successive generation, that is subject to selection. Phenotypic noise was modeled as a binomial distribution (*N* = 16) with an expected standard deviation of 4.0 mS/cm^2^. The *N* value for the noise distribution was selected by running successive simulations to obtain a best fit to the experimentally observed conductance value. Note that the distributions shown in (B) and (C) come from the same simulation run. D. Evolution of potassium conductance phenotype under selection for reduced action potential duration using the fitness function shown in Panel G. The average potassium conductance within the population remains stable over time (10000 generations, population = 5000). The blue line is the average value of ten simulation runs and five independent runs are shown in light grey on the same graph. The average final conductance value was 35.6 ± 3 mS/cm^2^ (mean ± S.D., n = 10). E. Histogram of a representative final phenotype distribution. In this simulation no phenotypic noise was used so that the final channel phenotype maps directly from the final cis-regulatory genotype. F. The fitness function used in (A, B and C). This was based on the minimum potassium conductance required to produce Type 3 firing properties, i.e. suppress repetitive firing in response to a sustained depolarizing current injection (see Fig 3A and 3B). G. Fitness function used in (D and E). Action potential duration as a function of potassium conductance (see Fig 3D) was inverted, scaled and then combined with a fitness cliff at 3 mS/cm^2^, the value at which action potential conduction fails.

Two mechanisms can account for the observed potassium conductance values. First, the discontinuity in axonal excitability at 24 mS/cm^2^ (Fig 3A and 3B) may produce a fitness cliff. Incorporation of this fitness cliff into the model by itself results in selection of a potassium conductance of 28.6 ± 0.7 mS/cm^2^, which still falls below the experimental value. Addition of a phenotypic noise distribution can maintain a stable potassium conductance (36.2 ± 0.5 mS/cm^2^) that is close to the experimental value (Fig 7A and 7C). Under these conditions there is reduced variation in the underlying potassium channel cis-regulatory genotype (Fig 7B).

Alternatively, it is possible that there is positive selection for a relatively short action potential duration (Fig 3D). A fitness function (Fig 7G) that incorporates the dependence of action potential duration on potassium conductance with appropriate scaling can also result in selection of an average potassium conductance close to the observed value (35.6 ± 3 mS/cm^2^). As seen for the sodium conductance, a fitness function with a shallow slope results in greater variation in the cis-regulatory phenotype both over time (Fig 7D) and within the population (Fig 7E).

### Concurrent Selection for Both Sodium and Potassium Conductances

Natural selection is conservative and incremental so that channel expression levels can remain stable across wide phylogenetic distances [12]. Nonetheless, specific channel expression levels can also evolve relatively quickly when selection pressures change [12,13]. How sodium and potassium conductances might evolve from a range of starting values towards the observed values maintained by purifying selection was examined.

**Fig 8:**
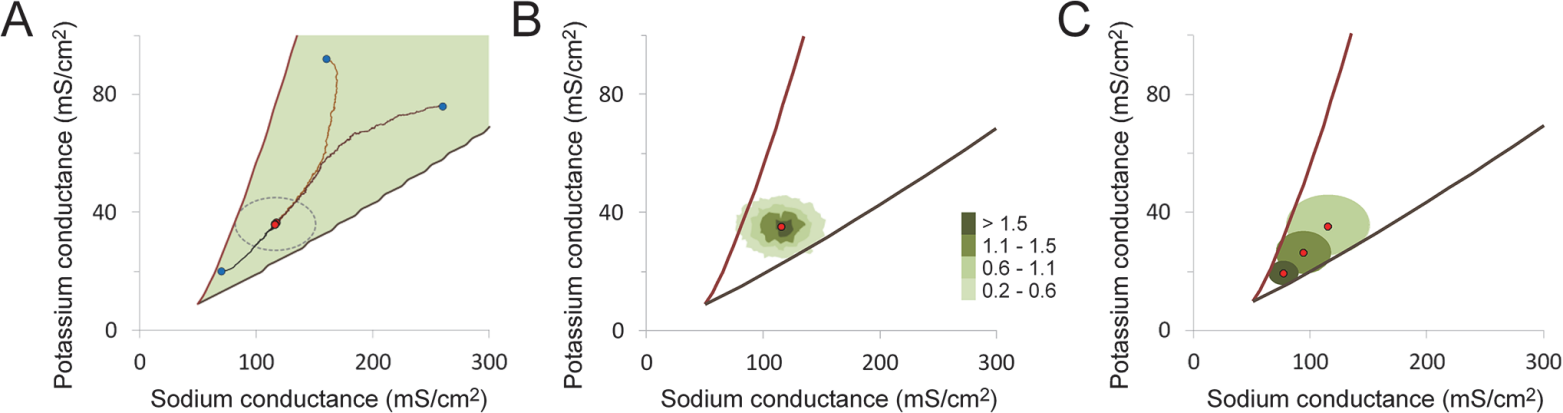
Stability of the end-point for sodium and potassium channel expression from multiple starting points. A. The region of sodium and potassium conductance parameter space that supports action potential conduction at 26°C and Type 3 firing properties (filled green area) bounded by two fitness cliffs (red and brown lines). Three different populations started from a different combination of average sodium and potassium conductance values (blue dots) that converged over successive generations to stable final values (red dots, overlapping symbols). The average final conductance values were G_Na_ = 116.5 ± 0.6 mS/cm^2^ and G_K_ = 36.0 ± 0.3 mS/cm^2^ (mean ± S.D., n = 3). The colored traces (black, orange and brown) mark the path of the average conductances over 4000 generations for each population, from its starting point to the stable final phenotype. The phenotypic noise used in the simulations had an expected standard deviation of 17.3 mS/cm^2^ for the sodium conductance and 4.5 mS/cm^2^ for the potassium conductance. These values were previously selected by iteration in order to fit the experimentally observed conductance values. The ellipse (dotted grey line) is centered on the average conductances and the length of the two axes correspond to 4 times the S.D. of the phenotypic noise used in both dimensions. B. The distributions of conductance phenotype for the final population at the end of a representative simulation run. A contour plot of 2D histograms of sodium and potassium conductance combinations shows the final phenotype distribution. Color bar indicates the number of individuals found in each bin, expressed as a percentage of the total population. Bins with fewer individuals than 0.2% of the total population were blanked. The red dot indicates the average conductance values for the population. The variation in channel expression levels within this population reflects a combination of variation in the sodium and potassium channel cis-regulatory genotype as well as the contribution of phenotypic noise. It is this distribution of channel protein expression values, which varies slightly with each successive generation, that is subject to selection. C. Average conductance values for the population increase as the population variation increases. Three simulations are shown. The three different final average conductance values (red dots) are shown with their corresponding population variation (solid ellipses, distinguished as three different shades of green). The changes in population variation were driven by changing the phenotypic noise values. Final conductance values for the three representative individual populations were: G_Na_ = 77.5 ± 6.1, 94.3 ± 11.7, 115.6 ± 18.0 mS/cm^2^ and G_K_ = 19.3 ± 2.0, 26.4 ± 3.5 and 35.7 ± 5.1 mS/cm^2^ respectively (mean ± S.D., N = 5000). The filled ellipses are centered on the average conductances and the length of the two axes correspond to 4 times the S.D. for that population.

For this simulation it was assumed that only those combinations of conductance values that could sustain axonal conduction up to 26°C and had Type 3 firing properties resulted in successful phenotypes (Fig 8A, green area). Phenotypic noise distributions for both the sodium and potassium conductance that could maintain average conductance values close to the experimental values over infinite generations were first fitted by iteration, similar to that described for the simulations shown in Fig 5B and 5C and Fig 7B and 7C. Simulations were then started from combinations of higher or lower average sodium and potassium conductance values. Over successive generations average, conductance values of the population would reliably converge from any higher or lower combination of starting conductances (Fig 8, blue circles) towards to the experimental conductance values (Fig 8, red circles), assuming that the starting population began within the set of successful phenotypes. At higher starting values, the accumulating effects of mutation cause the average conductance values to fall towards the final stable values. At low starting points, variation in channel expression causes a significant number of individuals to fall over the fitness cliffs, favoring the selection of individuals with higher conductance values. The effect of this positive selection gradually diminishes as the average conductances in the population converge towards the experimental values and a mutation-selection balance is established. The variation in channel expression values within the population (Fig 8B) reflects a combination of variation in the underlying cis-regulatory genotype of the two channels combined with variation due to phenotypic noise.

Increasing dispersion of population conductance values causes a systematic increase in average channel expression levels (Fig 8C). This illustrates an interesting trade-off between energy efficiency and robustness. The most energy efficient phenotypes (lowest average channel expression levels) are found in the population with the least phenotypic variation (Fig 8C). However, this population is also the most fragile, since a higher proportion of its individuals are found in close proximity to one of the two fitness cliffs. With increasing population variation in channel expression levels more individuals become robust, in the sense that their phenotype lies further and further away from the fitness cliffs and are, therefore, decreasingly likely to fail, even under unusually adverse circumstances.

## Discussion

The model of gene regulatory evolution described in this paper has two key properties.

1. The accumulating effects of mutation on gene regulatory function will cause expression of specific voltage-gated channel genes to fall to near zero in the absence of positive selection for channel expression.
2. In the presence of positive selection, channel expression levels will fall to the lowest level consistent with the selection criteria, due to the effect of accumulating mutations on gene regulatory function.

Both of these properties are expected and have been observed experimentally [14]. The linkage between the regulatory evolution model and the model of axonal action potential conduction is based on the relationship between physiological performance and evolutionary fitness. Despite the critical importance of this relationship in shaping much of biological function, it has only been studied experimentally in a few very favorable organisms [14]. Modeling is the only way to study this issue in most organisms. We consider two basic conditions: regions of discontinuity in the relationship between channel expression levels and physiological performance that might result in fitness cliffs and regions where this relationship is continuous and potential increases in fitness are the result of quantitative changes in physiological performance.

The performance of cellular electrophysiological systems can be a highly non-linear function of voltage-gated ion channel expression levels, resulting in distinct discontinuities in function [47,48]. For the squid axon, there are two clear functional discontinuities: the success or failure of the axon to transmit an action potential and the transition between Type 3 and Type 2 excitability (Figs 2 and 3). Discontinuities in physiological performance are likely to contribute disproportionately to fitness, resulting in ‘fitness cliffs’, borders in channel expression parameter space between regions of relatively high and relatively low fitness.

Failure of action potential conduction in the giant axon will have a significant effect on predator-escape and prey-capture behavior in the squid [39,40] and it is reasonable to assume that this discontinuity in physiological performance will have a large effect on fitness. The effect of the transition between Type 3 and Type 2 excitability on fitness is more ambiguous. Type 3 excitability is conserved in cold water squid [21], suggesting that this property is critical for the performance of the squid motor circuit. Type 3 neurons allow finer temporal precision than do repetitively firing Type 2 neurons and are important for fine-tuning the response to synaptic input and ensuring one-to-one mapping between the firing of pre- and post-synaptic cells [24]. The giant axon is the main site of synaptic integration leading to action potential generation so the subthreshold behavior of the axon can be a critical feature in tuning overall motor circuit performance. A low noise axon may be of particular importance due to the unusually direct linkage between axon firing and key behavioral responses [40,46].

Incorporation of fitness cliffs associated with these two discontinuities in physiological performance into the genetic regulatory model does not by itself result in selection of appropriate channel conductance values (Fig 4B). There are two sources of noise in the system that can affect channel expression independent of variation in the cis-regulatory function of the sodium and potassium channels. First, because the squid are drawn from a wild population comprised of genetically diverse individuals, there will be genetic variation in background genes, both their expression and function, which can act in *trans* to produce variation in the expression of the two channel proteins. Second, even within genetically identical individuals, protein expression levels can still be subject to multiple sources of noise, particularly for a population such as the squid that develops and lives under widely varying environmental conditions [35,36]. The model lumps these sources of variation into a single noise source termed ‘phenotypic noise’. The combination of fitness cliffs with phenotypic noise can produce good agreement between the model and experimental observation. This outcome leads to the simple hypothesis that the selection of channel expression values can, in many cases, be explained solely by the requirement that: a) electrophysiological function does not fail under the most extreme conditions that might be routinely encountered and b) physiological function must be sufficiently robust that it does not fail in the presence of the multiple sources of noise typically found in a natural population. The ability to avoid failure under all conditions normally encountered, including the effects of environmental and intrinsic noise, may be a sufficient explanation for the selection of channel conductance levels in the squid axon, and possibly other electrophysiological systems. This very simple mechanism should probably be considered the null hypothesis in any discussion regarding how channel expression levels might be established.

It was originally suggested that sodium conductance levels were selected in order to optimize conduction velocity [1], but this was refuted on the basis of the large difference between the experimentally observed sodium conductance and the much higher value required to produce the maximum conduction velocity [3]. It was subsequently argued that the sodium conductance was selected to minimize the metabolic cost of action potential generation [4]. The sodium conductance does lie close to the minimum metabolic cost value (Fig 2C) but there is an alternative explanation for this outcome: the cumulative effects of mutation on sodium channel gene regulatory function will produce a similar result. Since mutation is an intrinsic property of the genetic regulatory system, this would seem to be a sufficient explanation for this observation.

In general, arguments regarding optimality are complicated by the effect of mutation. Even if the sodium conductance was selected primarily to maximize conduction velocity any discrepancy between the optimal and experimental values could arise from a mutation-selection balance (Fig 5C). Because the mutation rate is always non-zero, this balance point will always fall below the optimal conductance value for maximum conduction velocity. The observation of a discrepancy between the optimal and observed conductance values [3] is not, in itself, a reason to reject a contribution of this property to fitness.

For the potassium channel, selection for either maximum conduction velocity or minimum metabolic cost will produce much lower conductance values than are observed experimentally (Fig 2C and 2E). The other continuous property that might contribute to fitness is action potential duration, which can be under strong selection in some systems [12]. If action potential duration is subject to selection in the squid axon, this would most likely be related to its large effect on calcium flux and neurotransmitter release in the nerve terminal [49].

In summary, a fitness cliff in combination with phenotypic noise can be a sufficient explanation for the selection of sodium conductance levels, because of the likelihood of a very strong effect of action potential failure on fitness. A similar mechanism may also explain the selection of potassium channel levels. The linkage between axonal excitability pattern and fitness is less certain, however, so the possibility that quantitative variation in a trait such as action potential duration may underlie the selection of potassium conductance levels cannot be excluded.

The results reported here are relevant to several current issues in the literature. It has been suggested that there is strong positive selection for the channel expression levels that maximize metabolic efficiency in neurons and that this may even be a major organizing principle in neuron evolution [4,43,44]. In general, the effect of mutations on gene regulatory function will cause voltage-gated ion channel expression levels to fall towards the lowest levels consistent with any positive selection criteria acting on the system. This process will automatically tend to produce an energy efficient system, even if there is no positive selection for energy efficiency. It might be argued that the squid axon is different to other electrophysiological systems, since it fires relatively infrequently and has relatively inefficient channel proteins [43,50], but the effects of mutation on the regulation of channel expression will be similar in other systems since the basic principles of gene regulation are highly conserved across broad phylogenetic distances [30]. While this does not imply that energy efficiency is not an important constraint for nervous system evolution, it does complicate any arguments based on the observation of low levels of voltage-gated ion channel expression, since there is a more fundamental explanation for this property, it is a consequence of mutation within the genetic regulatory system. To convincingly demonstrate that minimization of energy utilization is a critical constraint on the evolution of voltage-gated ion channel expression levels, it is necessary to demonstrate that some other function, that is also important, is compromised in order to maximize energy efficiency, i.e. demonstrate that there are tradeoffs associated with the maximization of energy efficiency. This is particularly important because the opposite has been shown, metabolic efficiency can be compromised in order to ensure robust high-frequency firing [51], suggesting that energy efficiency is not an over-riding constraint.

Much has been made of the fact that voltage-gated ion channel proteins turn over frequently and it has been suggested that electrically excitable cells require unique activity-dependent homeostatic regulatory mechanisms in order to maintain the stability of channel expression and electrical function [52,53,54]. Models to describe activity-dependent homeostasis have been based on internal calcium sensing to provide feedback about electrical activity [52,53,54]. These models have several inherent problems.

First, in the years since the original description of these models, limited experimental evidence has emerged supporting the idea that biological calcium sensors are capable of the sophisticated frequency dependent calcium sensing necessary for these models to control even a small number of independent genes. Second, genetic studies have shown that a much larger number of genes contribute to electrical function and calcium handling [55] than the relatively small number (seven) of channels described in these models, suggesting that even if frequency dependent calcium sensors did exist, only a small subset of the total set of genes that determine electrical function could be controlled in an independent graded fashion using such a mechanism. The expression levels of the majority of the relevant genes would still have to be established by regulatory evolution and establishment of mutation-selection balances, as described in this paper. Third, even though homeostatic control is limited to a small subset of genes, these models have high failure rates (14%) [54]. In a biological system, failure rates of this magnitude would normally be eliminated relatively quickly by natural selection, in favor of more reliable systems.

A related issue is the suggestion that the individual variation in voltage-gated channel expression levels in identified electrically excitable cells necessarily reflects the action of homeostatic regulatory mechanisms [53,56]. There is, of course, significant individual variation in the expression of most genes and much of this variation can be shown to have a genetic basis [57,58]. And, as noted above, even among genetically identical individuals there can be considerable variation in protein expression [35,36]. The squid is an outbred population that will have considerable genetic diversity and because the animals grow in the wild, there will be large individual differences in developmental and life history, adding another layer of noise to the system. As a consequence, considerable individual variation in protein expression levels in the axon would be expected, without invoking any other mechanisms. Given the existence of these basic mechanisms for generating individual variation in channel protein expression levels, there is no absolute requirement to conclude that homeostasis plays a fundamental role in generating individual diversity in conductance values in this particular system. It is likely that this is more generally true.

Finally, it has been demonstrated that a broad range of channel expression levels can produce similar electrophysiological function [48,59], suggesting that there can be large, nominally neutral spaces for channel expression values. A large isofunctional parameter space is also seen for the squid axon (Fig 3B). Importantly, however, only a relatively small region of this functionally equivalent parameter space will normally be occupied in vivo due to the establishment of a balance between the effects of mutation and selection on gene regulatory function within the population (Fig 8B). Over the course of evolution channel expression levels will trend towards the lowest levels compatible with whatever positive selection criteria are acting on the system and will remain stable at those levels until the selection criteria change (Fig 8A). Individual variation in channel expression levels means that in natural systems average conductance values will generally lie at some distance from any boundaries between regions with different function to ensure robustness (Fig 8C). Small islands of isofunctionality in parameter space [48] will not be viable.

One limitation of our model is that we only explicitly consider one form of regulatory evolution, cis-regulatory evolution of the two channel genes the directly contribute to electrical excitability in the axon. The evolution of the expression or function of many other genes, as well as the evolution of post-transcriptional regulatory mechanisms, can also affect expression of these channels. The entire regulatory apparatus can be thought of as a set of information. Mutations will, on average, be more likely to degrade this information than enhance it, i.e. entropy will increase over time in the absence of selection. As a consequence, if selection for a given channel is relieved for some reason the end result will be a loss of channel expression over time, as seen in our simplified model. Like most proteins [9,14], voltage-gated channels appear to be predominantly transcriptionally regulated [37,60,61,62] so the focus on transcriptional mechanisms is a reasonable first approximation, but clearly regulation of channel expression is more complex.

The combination of population genetics and cellular electrophysiology models can be a powerful tool to gain insight into the evolution of electrically excitable cells and the trade-offs that may be involved in this process. In the past, there has been a tendency to assume that a single electrophysiological property has been optimized during the course of evolution [1,4,44]. This view does not account for either the inherent limitations of the evolutionary process or for the complexity of the interacting and potentially conflicting constraints that can contribute to fitness.
